# Bootstrapping phylogenies inferred from rearrangement data

**DOI:** 10.1186/1748-7188-7-21

**Published:** 2012-08-29

**Authors:** Yu Lin, Vaibhav Rajan, Bernard ME Moret

**Affiliations:** 1 Laboratory for Computational Biology and Bioinformatics, EPFL, EPFL-IC-LCBB INJ230, Station 14, CH-1015 Lausanne, Switzerland

**Keywords:** Bootstrap, Jackknife, Phylogenetic reconstruction, Rearrangement, Gene order, Comparative genomics

## Abstract

**Background:**

Large-scale sequencing of genomes has enabled the inference of phylogenies based on the evolution of genomic architecture, under such events as rearrangements, duplications, and losses. Many evolutionary models and associated algorithms have been designed over the last few years and have found use in comparative genomics and phylogenetic inference. However, the assessment of phylogenies built from such data has not been properly addressed to date. The standard method used in sequence-based phylogenetic inference is the bootstrap, but it relies on a large number of homologous characters that can be resampled; yet in the case of rearrangements, the entire genome is a single character. Alternatives such as the jackknife suffer from the same problem, while likelihood tests cannot be applied in the absence of well established probabilistic models.

**Results:**

We present a new approach to the assessment of distance-based phylogenetic inference from whole-genome data; our approach combines features of the jackknife and the bootstrap and remains nonparametric. For each feature of our method, we give an equivalent feature in the sequence-based framework; we also present the results of extensive experimental testing, in both sequence-based and genome-based frameworks. Through the feature-by-feature comparison and the experimental results, we show that our bootstrapping approach is on par with the classic phylogenetic bootstrap used in sequence-based reconstruction, and we establish the clear superiority of the classic bootstrap for sequence data and of our corresponding new approach for rearrangement data over proposed variants. Finally, we test our approach on a small dataset of mammalian genomes, verifying that the support values match current thinking about the respective branches.

**Conclusions:**

Our method is the first to provide a standard of assessment to match that of the classic phylogenetic bootstrap for aligned sequences. Its support values follow a similar scale and its receiver-operating characteristics are nearly identical, indicating that it provides similar levels of sensitivity and specificity. Thus our assessment method makes it possible to conduct phylogenetic analyses on whole genomes with the same degree of confidence as for analyses on aligned sequences. Extensions to search-based inference methods such as maximum parsimony and maximum likelihood are possible, but remain to be thoroughly tested.

## Background

Large-scale sequencing of whole genomes has enabled the inference of phylogenies based on the evolution of genomic architecture, under such events as rearrangements, duplications, and losses. Many evolutionary models and associated algorithms have been designed over the last few years and have found use in comparative genomics and phylogenetic inference (see [1-3]). However, the assessment of phylogenies built from such data has not been properly addressed to date. The standard method used in sequence-based phylogenetic inference is the bootstrap [4,5]. It relies on the presence of a large number of homologous characters that can be resampled; yet in the case of rearrangements, the entire genome is a single character. Alternatives such as the jackknife suffer from the same problem, while likelihood ratio tests [6,7] cannot be applied in the absence of well established probabilistic models. Two preliminary approaches have been proposed, one based on the jackknife [8] and one based on random perturbations [9], but both fall short of the performance standard of the bootstrap on sequence data.

We describe a novel approach to the assessment of distance-based phylogenetic inference from whole-genome data. Our approach restates the main characteristics of the jacknife and bootstrap in terms of noise modeling, itself a longstanding approach to robustness assessment in engineering.A number of new resampling methods are discussed along with extensive experimental testing, in both sequence-based and genome-based frameworks. We show empirically that our new approaches provide bootstrap support values that are as acceptable as those provided by the classic bootstrap for sequence data. (While the systematics community has long used the bootstrap as its reference method and has gained confidence in its use, no systematic experimental study of the approach had been conducted; our results fill this gap for distance-based methods and confirm the validity of phylogenetic bootstrapping). Finally, we test our approach on a small dataset of mammalian genomes, verifying that the bootstrap values match current thinking about support values of the respective values obtained from sequence-based studies. The focus on distance-based methods is due in part to simplicity and convenience: by reducing the input genomes to a distance matrix, these methods not only simplify the characteristics of the input data, but also make it straightforward to compare our method with methods for sequence-based inference. The focus is also due in part to two other characteristics of distance-based methods: they are very efficient compared to optimization searches such as maximum parsimony and maximum likelihood; and they remain the most commonly used in routine phylogenetic reconstruction—neighbor-joining [10] and minimum evolution [11] account for nearly half of the citations to phylogenetic methods. But most of all the focus is justified by a unique characteristic of whole-genome data: under a model of rearrangements, duplications, and losses, it is possible to compute very precise maximum-likelihood estimates of the true evolutionary distance, as we have a shown in a series of papers [12-15]. Moreover, such distance estimates can be extended to take into account non-uniform distributions of the rearrangements (such as a preponderance of events affecting short segments of the genome), whereas we have almost no results about the combinatorial models of rearrangements when such rearrangements are not uniformly distributed. Finally, we have been able to extend this bootstrapping approach to inferences made under the Maximum Parsimony or Maximum Likelihood criteria, although the quality of the assessment provided by our approach for such settings remains to be established.

We briefly review the bootstrap and jackknife approaches, as well as relevant characteristics of rearrangement data and of distance-based methods for phylogenetic inference.

### Bootstrap and jackknife

Quenouille [16] introduced the idea of jackknifing, which was further developed by Tukey [17], who gave it its name. Bootstrapping was introduced by Efron [18] and Felsenstein proposed bootstrapping for phylogeny reconstruction [5]. There are several expositions on these estimation methods at different levels of mathematical detail [4,19-22], while Soltis and Soltis [23] and Holmes [24] give surveys of bootstrapping in phylogeny reconstruction.

Given *n* data points *X* = {*x*_1_,…,*x*_*n*_} and a statistical estimator *E*(*x*_1_,…,*x*_*n*_), a *bootstrap replicate* is a fictional dataset $Y=\{y_{1}^{*},\ldots ,y_{n}^{*}\}$ constructed by sampling with replacement from *X*. From each such fictional dataset a value of the estimator *E* can be obtained. The key idea of bootstrapping is that the distribution of values thus obtained closely matches the original distribution of *E* and can be used to estimate the confidence limits on the estimator. The advantage of the method lies in its applicability to arbitrary and complicated estimators that may be analytically intractable [4,20].

In phylogeny reconstruction, the standard bootstrap for sequence data [5,25] samples columns with replacement from a multiple sequence alignment to create a new alignment matrix of identical dimensions. Thus each bootstrap replicate contains the same number of species and the same number of columns per species, but some columns from the original alignment may be duplicated and others omitted. Each column can be viewed as a variable that is drawn from a space of 4^*s*^ possible outcomes at each site—assuming nucleic acid sequence data with *s* species and neglecting insertions, deletions, and ambiguity codes. From each replicate, a tree can be reconstructed using any of the available reconstruction techniques (such as distance-based methods, maximum parsimony, or maximum likelihood). The tree thus obtained from a single bootstrap replicate is a *bootstrap tree*. Many bootstrap trees are generated through repeated sampling and the *bootstrap score* (or *support*) of a branch in the inferred tree is computed as the proportion of the bootstrap trees that contain this branch (viewed as a bipartition of leaves). Soltis and Soltis [23] and Holmes [24] discuss the pros and cons of the approach in phylogeny reconstruction. A *jackknife* leaves out one observation at a time, thus creating a sample set *X*_(*i*)_ = {*x*_1_,…*x*_*i*−1_*x*_*i* + 1_,…,*x*_*n*_}. The estimator can be calculated on this new sample. The jackknife often provides a good approximation to the bootstrap, but it fails when the estimator is not smooth; moreover, the number of distinct sample sets is limited to the number of observations. Shao *et al.*[26] found that the generalized “delete-*d*” jackknife works well in practice, even for non-smooth estimators; in this version, *d* (or some fixed percentage) of the observations are randomly chosen and omitted to create the new sample set. A special case is *parsimony jackknifing *[27] in which an observation is omitted with fixed probability of 1/*e* when creating a new sample set. In such a case, the expected size of the new sample set is (1−1/*e*) times the size of the original set, which corresponds to a modified bootstrapping procedure in which, after sampling, duplicate samples are not added to the new sample set. No systematic comparison of these methods has been conducted in the context of phylogeny reconstruction. Felsenstein [5] hinted at the equivalence of support values from classical bootstrapping and from 50% jackknifing. Farris *et al.*[28] argued that 50% jackknifing deletes too many characters and does not allow one to maintain a useful relationship between group frequency and support; they advocated the use of parsimony jackknifing. Salamin *et al.*[29] compared bootstrapping and jackknifing in the context of maximum-parsimony reconstruction and reported that bootstrapping and 50%-jackknifing were comparable at confidence levels of 90% and higher. Finally, Mort *et al.*[30] compared bootstrapping with 50% and 33% jackknifing (with and without branch swapping) and reported that all three methods provide similar support values.

### Rearrangement data

Rearrangement data for a genome consists of lists of syntenic blocks (genes are an example) in the order in which they are placed along one or more chromosomes. Each syntenic block is identified by a marker, which is shared with all (or most) of its homologs in the genomes under study; for convenience, distinct markers are indexed arbitrarily from 1 to *n*. If every marker is shared and unique, the data is assumed to have been produced solely through rearrangements; otherwise, duplications and losses of syntenic blocks form another part of the evolutionary history. A chromosome (linear or circular) is represented by a signed permutation (linear or circular) of the markers’ indices; the sign represents the strandedness of the corresponding syntenic block. A genome is a collection of such permutations, one per chromosome. Note that the actual sequence content of a syntenic block is ignored at this level: it was used only to identify the block. Interest in this type of data comes in part from the hypothesis that large-scale structural changes to the genome are “rare genomic changes” [31] and thus may clarify distant or problematic relationships among organisms. For that reason, such data has been used in a number of phylogenetic studies—see [2] for references.

### Distance-based phylogeny reconstruction from rearrangement data

Distance-based reconstruction methods typically run in time polynomial in the number and size of genomes—and fast and accurate heuristics exist for those where the scoring function cannot be computed in polynomial time, such as least-squares or minimum evolution methods. Further, methods like Neighbor-Joining (NJ) [10] provably return the true tree when given true evolutionary distances. However, the distances that can be computed with sequence data are often far from the true evolutionary distances, particularly on datasets with markedly divergent genomes. The true evolutionary distance—the actual number of evolutionary events between the two genomes—is impossible to measure, but it can be estimated using statistical techniques. A statistical model of evolution is used to infer an estimate of the true distance by deriving the effect of a given number of changes in the model on the computed measure and (algebraically or numerically) inverting the derivation to produce a maximum-likelihood estimate of the true distance under the model. This second step is often called a *distance correction* and has long been used for sequence data [32] as well as, more recently, for rearrangement data [33-35]. For multichromosomal genomes, we described such an estimator assuming equal “gene” content [13]. As rearrangement data is typically given in terms of syntenic blocks rather than genes, and as syntenic blocks are often unique, the limitation to equal gene content is not severe. Moreover, we recently refined our estimator to include gene duplication and loss events [15].

## Robustness estimation for trees reconstructed from rearrangement data

Providing bootstrap support scores is standard practice in phylogenetic reconstruction from sequence data. However, the classic bootstrap cannot be applied directly to rearrangement data because the collection of permutations forms a single character—a single rearrangement or duplication can affect an entire permutation. In the world of sequence data this is equivalent to an alignment with a single column, albeit one where each character can take any of a huge number of states. Two approaches have been suggested in the past, but do not match the accuracy of the classic bootstrap. One, proposed by Shi *et al.*[8], is a jackknifing technique; the second, proposed by us, uses perturbations in the permutations with known effects on the distance [9].

We design different methods for rearrangement data and devise analogous methods for sequence data (if they do not exist) and vice versa. We study their behavior with both kinds of data with the aim of developing a method for rearrangement data that is as successful as the classic bootstrap is for sequence data. For a method *M* that operates on sequence data, we denote by *M*^∗^ the corresponding method for rearrangement data; we use regular font to denote existing methods, bold font to denote the new methods described in this paper.

The methods we present here for rearrangement data rely on our distance estimator [13] and so must be used with distance-based reconstruction methods. Our distance estimator computes the estimated true distance between two multichromosomal genomes, replying on statistical inference of only the number of shared adjacencies and the number of linear chromosomes. This limited view of the input data is crucial, as many of the sampling approaches we describe below do not produce valid genome permutations (e.g., because of additional copies of adjacencies), yet still allow us to tally the number of linear chromosomes and of shared adjacencies.

Our robustness estimator based on distance perturbation [9], hereafter denoted BP*, permutes each leaf genome through a (randomly chosen) number of random rearrangements, estimates the new pairwise distances, then subtracts from each pairwise estimate the number of rearrangement operations applied to each of the two genomes. The number of operations applied to each genome is chosen from a Gaussian distribution, and so, for each genome, is potentially different. If *x* operations are applied to leaf *i* to yield leaf *i*^*′*^ and *y* operations are applied to leaf *j* to yield leaf *j*^*′*^ (where leaves *i* and *j* are in the inferred tree and leaves *i*^*′*^ and *j*^*′*^ in the bootstrap), the expected distance between *i*^*′*^ and *j*^*′*^ is increased by (*x* + *y*) compared to the distance between *i* and *j*. To keep the expected pairwise distance after perturbation close to the distance between the corresponding pair of leaves before perturbation, we set the final (perturbed) distance *B*(*i*^*′*^*j*^*′*^)=*D*(*i*^*′*^*j*^*′*^)−(*x* + *y*). See [9] for more details. Thus BP* relies on additivity, a property likely to be respected with rearrangement data due to its huge state space. We can design an equivalent for sequence data: for each sequence, apply some random number of possible mutations under a chosen model, then estimate all pairwise distances under the same model, and finally subtract from that estimate the number of mutations applied in the perturbation step to each of the two sequences—a method we denote **BP**. **BP** is less reliable than BP*, as it is much more likely that some of the mutations used in the perturbations cancel each other or cancel some of the mutations on the edit path between the two sequences.

We can view the classical bootstrap for sequence data (hereafter denoted BC) in terms of noise generation. The original multiple sequence alignment gives rise to a distance matrix *D*. Each replicate dataset created by sampling columns with replacement from the alignment also gives its corresponding matrix *B* of perturbed pairwise distances. An entry of the replicate matrix corresponding to leaves *i* and *j* can thus be written as *B*(*i*,*j*)=*D*(*i*,*j*) + *N*(*i*,*j*) where *N*(*i*,*j*) denotes the perturbation in the distance introduced by the resampling. This noise parameter is hard to characterize exactly, but it leads us to define bootstrapping approaches based on producing increasingly refined estimates of the noise. (In that sense, BP* and **BP** attempt to shape the noise by returning to the underlying evolutionary process of rearrangement or mutation.)

Bootstrapping by adding Gaussian Noise (hereafter denoted **BGN**), adds Gaussian noise of mean 0 to each entry in the distance matrix. The standard deviation is empirically determined to match as well as possible the noise added by BC. Since the noise added during the sampling process in BC is not random, this is a very rough estimate, but a useful comparison point. In the replicate matrices produced by BC, the noise *N*(*i*,*j*) depends on the pairwise distance *D*(*i*,*j*), so the next step is to design a bootstrap method based on pairwise comparisons, hereafter denoted **BPC**. The bootstrap matrix *B*(*i*,*j*) for **BPC** is constructed by calculating the perturbed pairwise distance for each pair: for each pair of sequences *i*,*j*, we construct a new pair of sequences *i*^*′*^,*j*^*′*^by sampling columns with replacement, where each column has only two characters and set *B*(*i*,*j*)=*D*(*i*^*′*^,*j*^*′*^).

An equivalent method BPC* can be designed for rearrangement data, albeit with some complications. Since our distance estimator relies on the number of shared adjacencies, a natural choice is to sample adjacencies in the genome. While the evolution of a specific adjacency depends directly on several others, independence can be assumed if we assume that once an adjacency is broken during evolution it is not formed again—an analog of Dollo parsimony, but one that is very likely in rearrangement data due to the enormous state space. For each pair of genomes *i*,*j*, we construct two new pairs of genomes. We sample adjacencies from genome *i* with replacement and use only these adjacencies to compute the distance *D*_1_(*i*,*j*) of leaf *i* to leaf *j*. (Note that some adjacencies may be overcounted and some omitted.) Then we sample adjacencies from genome *j* with replacement and use only these adjacencies to compute the distance *D*_2_(*i*,*j*) of leaf *j* to leaf *i*. Finally, we set *B*(*i*,*j*)=(*D*_1_(*i*,*j*) + *D*_2_(*i*,*j*))/2.

The noise *N*(*i*,*j*) may depend not just on the pairwise distance *D*(*i*,*j*), but also on other distances in the tree, since BC samples columns with replacement for all leaf sequences *at once*. The next step in modeling *N*(*i*,*j*) is thus to sample from all adjacencies (including telomeres). The total number of possible adjacencies (including telomeres) for *n* syntenic blocks is roughly 2*n*^2^, but in a given genome there are at most 2*n* adjacencies and each adjacency conflicts with at most 4*n*other adjacencies. Thus, for large genomes, we may assume that adjacencies are independent (if rearrangements happen randomly), just as columns of an alignment are assumed to be independent in BC. We can now mimic closely the sampling procedure of BC in a rearrangement context, producing procedure BC*. From the list of all possible adjacencies, BC* samples with replacement to form a collection of adjacencies; only adjacencies in this collection are then considered in counting the number of shared adjacencies and then estimating the true evolutionary distances between genomes. (Note that some shared adjacencies are counted more than once due to the sampling with replacement).

We know that classical bootstrapping (BC) is comparable in performance to parsimony jackknifing (which we denote PJ) in the sequence world. PJ is (asymptotically) equivalent to sampling with replacement (as in BC), but without overcounting, that is, when sampling gives a column that has been previously selected, it is not added to the replicate. Thus we can obtain the equivalent of PJ for rearrangement data, call it PJ*: selected adjacencies are not counted more than once for computing the number of shared adjacencies between leaves. Other versions of jackknifing are similarly easy to design. For instance, a *d%*−jackknife (*d*JK) omits *d%*of the columns to create a replicate, so, from the set of all adjacencies (in all the leaf genomes) a *d%*-jackknife (*d*JK*) deletes *d%*of the adjacencies at random and only the remaining adjacencies are used in estimating the true pairwise distances. In contrast, the previous jackknifing approach for rearrangement data, developed by Shi *et al.*[8], produces replicates by deleting syntenic blocks from the genome: a *d%*-jackknife, in their method, produces a dataset where *d%*of the markers are randomly deleted from all leaf genomes. The authors recommend setting *d*=40; we call the resulting method JG*. Note that our approach to jackknifing deletes adjacencies instead of markers.

In summary, we have designed a bootstrapping procedure, BC*, that closely mimics the classic bootstrap for phylogenetic reconstruction, BC, and jackknifing procedures, *d*JK* (including, as a special case, PJ*), that closely mimic the *d%*−jackknife (and parsimony jackknife PJ). Along the way, we have also designed less refined versions of bootstrapping and their equivalents for sequence data. In our experiments, we use all of these, plus JG*, the marker-based jackknifing approach of Shi *et al.*, plus BP*, our earlier approach based on introducing perturbations, in the permutations, of known effect on the distances [9]. A summary of all the methods can be found in Table 1.

**Table 1:** A summary of all the methods

**BGN**, BGN*	Bootstrap by adding Gaussian Noise to the distance matrix.	
**BPC**, BPC*	Bootstrap by Pairwise Comparisons: for each pair of sequences/genomes, sample	
	columns/adjacencies with replacement to compute distance.	
BC, BC*	Classical Bootstrap: sample columns with replacement to obtain replicate; sample	
	adjacencies with replacement to compute distance matrix.	
PJ, PJ*	Parsimony Jackknifing: choose each column with 1−1/*e* probablity to create replicate;	
	sample adjacencies with replacement and discard duplicates to compute distance matrix.	
dJK, dJK*	*d*%-JackKnife: Omit *d*% of columns at random to produce replicate; omit *d*% of adjacencies	
	at random to compute distance matrix.	
**BP**, BP*	Bootstrap by Perturbations: apply random mutations/rearrangements to get replicates.	
JG*	Jackknife Genes: Marker based jackknifing method of Shi *et al* for rearrangement data.	

## Experimental design

Our simulation studies follow the standard procedure in phylogeny reconstruction (see, e.g., [36]): we generate model trees under various parameter settings, then use each model tree to produce a number of true trees on which we evolve artificial genomes from the root down to the leaves to obtain datasets of leaf genomes for which we know the complete history. The sequences are evolved by random point mutations under the Kimura 2-parameter (K2P) model (see [32]) using various transition/transversion ratios; the permutations are evolved through double-cut-and-join (DCJ) operations chosen uniformly at random. (DCJ [37,38], has become the most commonly used model of rearrangement for multichromosomal data and is the rearrangement operator targeted by our distance estimator.) The resultant leaf sequences are without gaps, are of the same length and do not need further alignment. For distance-based reconstruction, the distances between leaf sequences are given by the standard distance estimate for the K2P model [32] and the tree is reconstructed with the Neighbor-Joining (NJ) [10] and FastME [11] algorithms. Unless stated otherwise, the tree-building algorithm used is Neighbor-Joining. We chose to use NJ (rather than the more accurate FastME) in most of our experiments to highlight the discriminating ability of the bootstrapping methods. For rearrangement data we reconstruct trees by computing a distance matrix using our DCJ-based true distance estimator [13] and then using this matrix as input to the tree-building method.

A model tree consists of a rooted tree topology and corresponding branch lengths. The trees are generated by a three-step process. We first generate trees using the birth-death tree generator (from the geiger library) in the software R [39], with a death rate of 0 and various birth rates (data shown below is for a rate of 0.001). The branch lengths in this tree are ultrametric (the root-to-leaf paths all have the same length), so, in the second step, the branch lengths are modified to eliminate the ultrametricity. Choosing a parameter *c*, for each branch we sample a number *s* uniformly from the interval [−*c*, + *c* and multiply the original branch length by *e*^*s*^ (we used various values of *c*; data shown below is for *c*=2). Finally, we rescale branch lengths to achieve a target diameter *D* for the model tree. (Note that the unit of “length” is one expected evolutionary operation—rearrangement or mutation, and the target diameter is the length of the longest path in the tree) Each branch length now represents the *expected* number of evolutionary operations on that branch. From a single model tree, a set of trees is generated for simulation studies by retaining the same topology and varying the branch lengths by sampling, for each branch in the tree, from a Poisson distribution with a mean equal to that of the corresponding branch length in the model tree.

Experiments are conducted by varying the number of syntenic blocks and the target diameter. We use trees with 100 leaves. Among the many parameter values tested we show the following representative settings: for sequence data, each leaf has 10,000 characters and the tree diameter is 20,000, while, for rearrangement data, we show the results on two sets of parameters, one where each genome has 1,000 markers and the tree diameter is 2,000 and another where each genome has 5,000 markers and the tree diameter is 15,000. For each setting of the parameters, 100 model trees are generated and from each model tree 10 datasets are created; we then average results over the resulting 1,000 trees. For each experiment we produce 100 replicates and thus 100 bootstrap trees from which to compute the bootstrap support of each branch.

A Receiver-Operator-Characteristic (ROC) curve is drawn for every method we investigate. In this plot, a point is a particular bootstrapping test, defined by its *sensitivity* and *specificity*; in the system of coordinates of our figures, a perfect test would yield a point at the upper left-hand corner of the diagram, with 100% sensitivity and 100% specificity. Define *E* to be the set of edges in the true tree and *T*_*t*_, for a threshold *t*, to consist of those edges in the inferred tree that are contained in more than *t%*of the bootstrap trees. Sensitivity is the proportion of true edges that are also in *T*_*t*_, |*T*_*t*_∩*E*|/|*E*|, while specificity is the proportion of edges in *T*_*t*_ that are true edges, |*T*_*t*_∩*E*|/|*T*_*t*_|. In our tests we use every fifth value in the range [0,100] as thresholds.

## Experimental results and analysis

**Figure 1:**
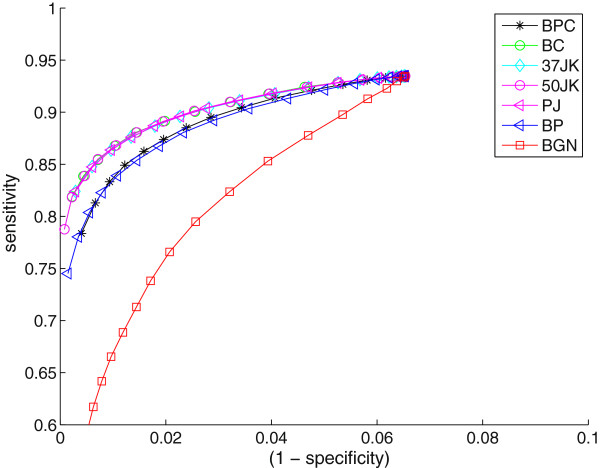
Bootstrapping methods for sequence data.

Figure 1 shows the ROC curves of the methods for sequence data, for 100 sequences of 10,000 characters each, and a tree diameter of 20,000. The four “reference” methods—50%-jackknifing (50JK), classical bootstrapping (BC), (1/*e*)*%*-jackknifing (37JK), and parsimony jackknifing (PJ)—are nearly indistinguishable and clearly dominate the others. The analogs of all the other methods developed for rearrangement data (**BP**, **BPC** and **BGN**) are clearly worse than the above four, with **BP** and **BPC** being comparable and the most primitive noise-shaping method, **BGN**, doing the worst.

**Figure 2:**
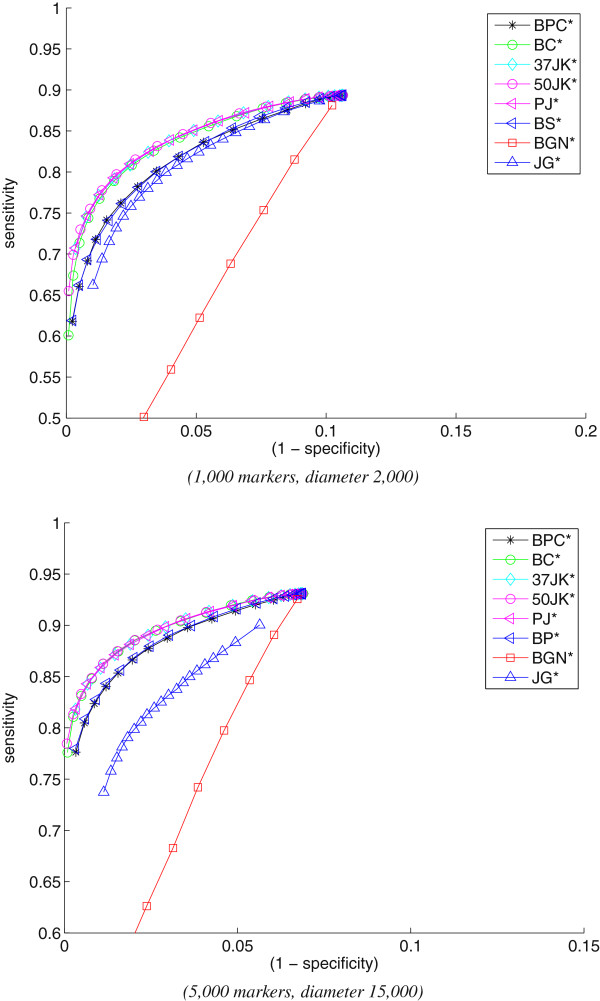
Bootstrapping methods for rearrangement data (using NJ).

Figure 2 shows the ROC curves for rearrangement data for different model conditions. The results follow the same pattern as for sequence data: BC*, PJ*, 50JK*, and 37JK* are nearly indistinguishable and clearly dominate all others. They are followed by BP* and BPC*, which are comparable, while the Gaussian noise approach, BGN*, again does the worst. JG*, the marker-based jackknifing technique of Shi *et al.*, is better than BGN*, but trails all other methods. The differences are particularly marked at very high levels of specificity; at 98% specificity, for instance, the top four methods retain nearly 90% sensitivity, but JG* drops to 80%. Very high specificity is the essential characteristic of a good bootstrap method.

**Figure 3:**
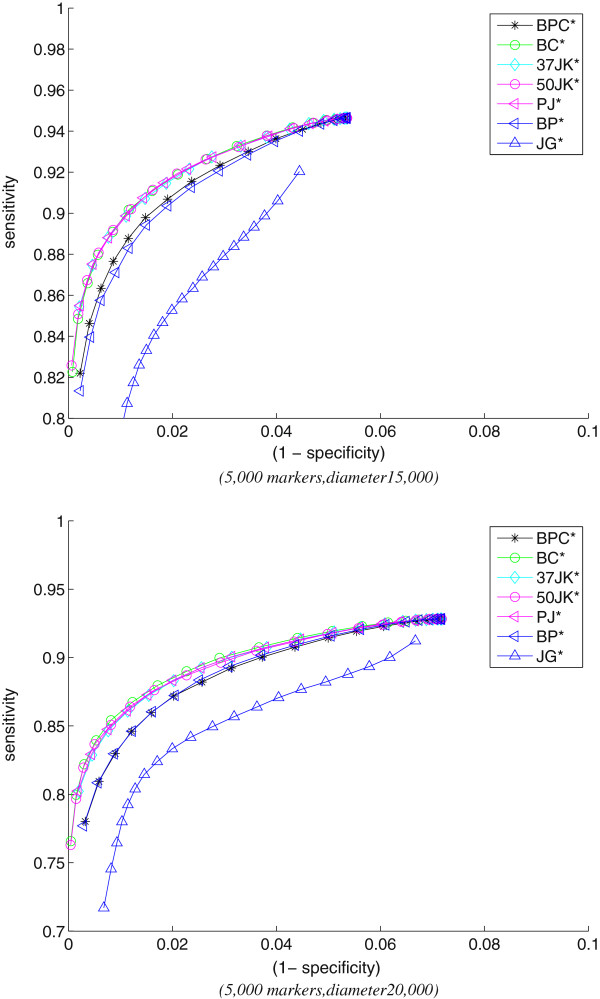
Bootstrapping methods for rearrangement data (using FastME).

Figure 3 shows the ROC curves for rearrangement data when FastME is used for tree reconstruction instead of NJ. We observe that the relative behavior of the bootstrap methods do not change: BC*, PJ*, 50JK*, and 37JK* perform equally well and dominate BP* and BPC*. Since the reconstructed trees using FastME are more accurate, the sensitivity of the top four methods at high levels of specificity are even higher compared to the sensitivity attained when NJ was used (Figure 2).

### A dataset of vertebrate genomes

**Figure 4:**
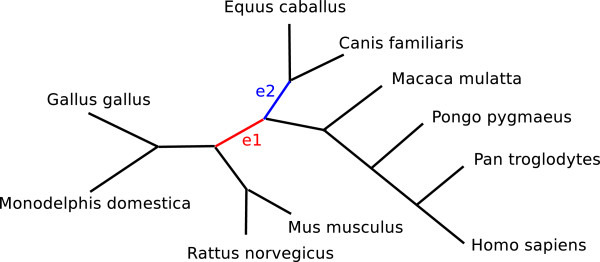
Inferred phylogeny of 10 vertebrates.

We also tested our bootstrapping methods on a real dataset: the genomes of 10 species from the Ensembl Mercator/Pecan alignments with 8,380 common markers. Four of these genomes (horse, chimpanzee, rhesus, and orangutan) are not well assembled: their draft genomes have nearly twice as many contigs as there are chromosomes—but the effect on our adjacency-based distance estimator is minimal, given the large number of markers. Figure 4 shows the inferred phylogeny and highlights the two edges with lowest bootstrap support (according to our BC* bootstrapping method). Based on previous studies [40-45] the edge *e*_1_ is uncertain: some studies place the primates in a clade with rodents, while others place them in a clade with the carnivores. Thus we would expect *e*_1_to receive the lowest support in the tree. BC* does give it the lowest support: 77% for *e*_1_ and 83% for *e*_2_. BP* gives low support values for both (49% for *e*_1_and 44% for *e*_2_), but fails to identify *e*_1_as the least supported edge, while JG* erroneously gives high support values to both (100% for *e*_1_ and 90% for *e*_2_).

## Conclusion

Our new approach for whole-genome data, based on the sampling of adjacencies, matches the classical bootstrap and parsimony jackknife approaches and thus provides the first reliable method for assessing the quality of phylogenetic reconstruction from such data.

In the process of testing various methods, we also confirmed past findings about the superiority of the phylogenetic bootstrap and of the parsimony jackknife. Our results clearly indicate that duplicate samples play no role in the process—parsimony jackknifing works at least as well and occasionally slightly better. Indeed, the best sampling strategy appears to be a random sampling of half of the characters. Given the very high computational cost of the bootstrap, using half the number of characters in sequence-based analyses appears a worthwhile computational shortcut, especially as it delivers even better results.

Our study focuses on distance-based methods, which reduce the collection of input genomes to a distance matrix. Our basic approach is to equate sampling characters in sequence data with sampling adjacencies in whole-genome data. Any reconstruction method that can handle such data can use this bootstrap procedure. Our reconstruction method is one such method since our distance estimator only counts the number of shared adjacencies between genomes and the number of linear chromosomes in each of them. Possible alternatives for methods (such as Maximum Parsimony) that are unable to handle such data include parsimony jackknifing and direct encoding of adjacencies into sequences. In parsimony jackknifing (PJ*), each original genome is represented by a set of contiguous regions in the bootstrap; if the reconstruction method can handle such inputs, then this is the best method. Encoding rearrangement data into sequences was proposed many years ago (see [46]) in two different versions (binary encodings and multistate encodings). In such methods, the input is simply a collection of (perfectly) aligned sequences and so the output can be assessed by the standard phylogenetic bootstrap. The early encodings fared poorly in comparison with MP methods (for rearrangement data), but a recent paper [47] suggests that a more complex encoding may overcome these problems.

## Competing interests

The authors declare that they have no competing interests.

## Author’s contributions

YL and VR contributed equally to this work. YL and VR conceived the idea, implemented the methods, and conducted the experiments. BMEM directed the project. YL, VR and BMEM collaborated closely in the elaboration of the project, the analysis of the data, and the writing of the manuscript. All authors read and approved the final manuscript.
